# Human Mitochondrial Hsp70 (Mortalin): Shedding Light on ATPase Activity, Interaction with Adenosine Nucleotides, Solution Structure and Domain Organization

**DOI:** 10.1371/journal.pone.0117170

**Published:** 2015-01-23

**Authors:** Paulo R. Dores-Silva, Leandro R. S. Barbosa, Carlos H. I. Ramos, Júlio C. Borges

**Affiliations:** 1 Institute of Chemistry of Sao Carlos, University of Sao Paulo, Sao Carlos, SP, P.O. Box 780, 13560–970, Brazil; 2 Institute of Physics, University of Sao Paulo, Sao Paulo, SP, 05508–090, Brazil; 3 Institute of Chemistry, University of Campinas—UNICAMP, P.O. Box 6154, 13083–970, Campinas, SP, Brazil; UMCG, NETHERLANDS

## Abstract

The human mitochondrial Hsp70, also called mortalin, is of considerable importance for mitochondria biogenesis and the correct functioning of the cell machinery. In the mitochondrial matrix, mortalin acts in the importing and folding process of nucleus-encoded proteins. The *in vivo* deregulation of mortalin expression and/or function has been correlated with age-related diseases and certain cancers due to its interaction with the p53 protein. In spite of its critical biological roles, structural and functional studies on mortalin are limited by its insoluble recombinant production. This study provides the first report of the production of folded and soluble recombinant mortalin when co-expressed with the human Hsp70-escort protein 1, but it is still likely prone to self-association. The monomeric fraction of mortalin presented a slightly elongated shape and basal ATPase activity that is higher than that of its cytoplasmic counterpart Hsp70-1A, suggesting that it was obtained in the functional state. Through small angle X-ray scattering, we assessed the low-resolution structural model of monomeric mortalin that is characterized by an elongated shape. This model adequately accommodated high resolution structures of Hsp70 domains indicating its quality. We also observed that mortalin interacts with adenosine nucleotides with high affinity. Thermally induced unfolding experiments indicated that mortalin is formed by at least two domains and that the transition is sensitive to the presence of adenosine nucleotides and that this process is dependent on the presence of Mg^2+^ ions. Interestingly, the thermal-induced unfolding assays of mortalin suggested the presence of an aggregation/association event, which was not observed for human Hsp70-1A, and this finding may explain its natural tendency for *in vivo* aggregation. Our study may contribute to the structural understanding of mortalin as well as to contribute for its recombinant production for antitumor compound screenings.

## Introduction

Human mortalin (also named mtHsp70, GRP75, HspA9 and PBP74) [1–4] is a highly conserved molecular chaperone of the Hsp70 family that is primarily found in the mitochondria. Depending on its localization and its binding partners, mortalin has been associated with several functions, such as anti-apoptosis; interaction with wild-type p53 in the cytoplasm reducing its transcriptional activity [5–7]; transportation of nucleus-encoded proteins to the mitochondrial matrix [8–11] and to different regions of the cell [7]; cellular protection [6, 12–14]; cell protection against oxidative stress and death [13, 15–17]; and import and translocation of cytosolic proteins by association with Hsp60 [18], among other functions [4]. Moreover, mortalin is the import motor that drives the preprotein import process and helps the folding of these proteins in the mitochondrial matrix [11, 19]. Due to their importance for protein homeostasis, Hsp70 proteins have been considered targets for the drug-based treatments for cancers [7, 20–22], misfolding diseases and protein folding disorders [23].

Mortalin presents similar structural elements as other Hsp70 proteins: an N-terminal ATPase domain (NBD) and a C-terminal peptide-binding domain (PBD). These two domains should be reciprocally controlled by a bidirectional heterotrophic allostery dependent on the presence of ATP/ADP on the NBD and a client protein bound to the PBD [22, 24]. An ATP-bound state in the NBD leads the PBD to achieve a low-affinity state with client proteins, whereas peptide binding to PBD in to the presence of a J-protein co-chaperone stimulates weak ATPase activity in the NBD, which leads to conformational changes in Hsp70, resulting in an enhancement of the affinity of the PDB for client proteins. The exchange of ADP for ATP in the NBD returns the PBD to a low-affinity state for client proteins, leading to its release [22]. The mammalian mitochondria also presents the main Hsp70 co-chaperones: 1) J-proteins (Hsp40), which should stimulate Hsp70 ATPase activity, and 2) two GrpE orthologous proteins, which should act as nucleotide exchange factors controlling the rate cycle of Hsp70 [22].

The mammalian mtHsp70 is also called mortalin due to its activity in the senescence and cellular death processes in rats, which present two mortalin isoforms, namely MOT1 and MOT2 [25, 26]. MOT2 has only two different amino acids in the PBD and is associated with cell immortality. Humans have only one mortalin orthologue, which is similar to MOT2 [7, 24]. Interestingly, mortalin is not exclusively a mitochondrial protein because approximately 30% is found in other cellular compartments [7, 27, 28]. It has been shown that human mortalin is involved in several cellular processes, may present important roles in Parkinson’s and Alzheimer’s diseases [7, 21] and is overexpressed in cancer [13, 29]. Based on these observations, there is widespread interest for the functional and structural study of mortalin and the assessment of its regulation by co-chaperones and ligands [7] because the study of this protein has been limited due to its self-aggregation when produced heterogeneously [11, 30–32]. Our search of the literature identified only one study on full-length recombinant human mortalin, which was produced in inclusion bodies and obtained through chemical refolding strategies for structural/functional characterization [33]. Nevertheless, it is well known that chemical refolding cannot be reliable for obtaining recombinant proteins with all of their structural signatures. In the case of recombinant human mortalin, the samples did not show Hsp70 signatures, and the chemical refolding approaches led to protein aggregation/precipitation [33]. A new human mitochondrial Hsp70 co-chaperone denoted Hsp70-escort protein (hHep1) was recently reported to act by preventing mortalin self-aggregation [32, 34–36]. Using a co-expression strategy with hHep1, Zhai et al. (2008) obtained recombinant human mortalin in its monomeric and active form [32], suggesting the reliability of this strategy.

To deepen the structure-function relationship of human mortalin, we obtained the recombinant protein in its soluble and functional state and compared it to human Hsp70–1A: a cytoplasmic Hsp70 counterpart. The hydrodynamic characterization indicated that mortalin was obtained in the monomeric state, noting the effectiveness of the induction and purification protocols developed. The spectroscopy data confirmed that human mortalin was obtained with secondary and tertiary structures characteristic of homologous Hsp70. The enzyme kinetics experiments indicated that mortalin has higher ATPase activity than human Hsp70–1A. Moreover, mortalin interacted with adenosine nucleotides with a micromolar dissociation constant. The small angle X-ray scattering data noted mortalin’s monomeric state in the testing conditions and allowed the generation of an *ab initio* model, indicating its slightly elongated shape. Interestingly, the thermal stability characterization showed that mortalin is composed of two domains, which are sensitive to adenosine nucleotide in the presence of Mg^2+^ ions. These experiments also indicated that mortalin undergoes aggregation/association in the first thermal-transition that was not observed during the unfolding of human Hsp70–1A, and this finding may help explain the natural tendency of the *in vivo* aggregation reported for mortalin. To the best of our knowledge, this study is the first to shed light on the structure of a functional human recombinant mortalin.

## Material and Methods

### Protein expression and purification

The recombinant human Hsp70–1A was expressed in the *Escherichia coli* BL21(DE3) strain as previously described [37]. The recombinant human mortalin was produced as described by Dores-Silva et al. [34] with some modifications. Summarily, to obtain human mortalin in soluble form, it was co-expressed with its co-chaperone hHep1. The cells co-transformed with pET23a::hHep1 and pET28a::Mortalin were grown at 37°C in LB medium containing 50 μg.mL^-1^ ampicillin and 50 μg.mL^-1^ kanamycin to A_600 nm_ of 0.7, at which point protein expression was induced by 0.2 mmol.L^-1^ IPTG. After 18 h of induction at 23°C, the cells were harvested by centrifugation for 20 min at 2,600 x g. For cell lysis, the pellet was resuspended in 50 mmol.L^-1^ Tris-HCl (pH 8.0) and 100 mmol.L^-1^ KCl (15 mL/200 mL of LB medium) and incubated with 5 U of DNAse (Promega) and 30 μg.mL^-1^ lysozyme (Sigma) for 60 min in ice. The pellet was then disrupted through two sonication steps and then centrifuged at 20,000 x g and 4°C for 30 min. The supernatant was filtered, subjected to Ni^2+^-affinity chromatography in 20 mmol.L^-1^ phosphate (pH 7.5) and 100 mmol.L^-1^ NaCl, and eluted in the same buffer containing 500 mmol.L^-1^ imidazole. Immediately after the elution, the material obtained was incubated with alkaline phosphatase (New England BioLabs) on ice for 4 h to eliminate any traces of adenosine nucleotide [37] and subjected to size exclusion chromatography with a Superdex 200 pg column (GE Healthcare Life Sciences) in TKP buffer (25 mmol.L^-1^ Tris-HCl, pH 7.5, 50 mmol.L^-1^ NaCl, 5 mmol.L^-1^ sodium phosphate, 5 mmol.L^-1^ KCl, and 2 mmol.L^-1^ β-mercaptoethanol) at a controlled temperature. The efficacy of the expression and purification processes was assessed by SDS-PAGE. The protein concentration was determined spectrophotometrically using the extinction coefficient calculated for mortalin under native conditions.

### Spectroscopy studies

Circular dichroism measurements were performed with a J-815 spectropolarimeter (Jasco Inc.) coupled to the Peltier system PFD 425S for temperature control. Mortalin was tested in TKP buffer at final concentrations between 5 and 10 μmol.L^-1^ in a 1-nm or 0.2-mm circular path-length cuvettes. The CD spectrum of mortalin in the presence of adenosine nucleotides (200 μmol.L^-1^) and/or Mg^2+^ (200 μmol.L^-1^) were also collected. The spectra were normalized to the mean residue ellipticity ([Θ]), and the protein secondary structure content was estimated using the CDNN Deconvolution program [38].

Thermal-induced unfolding was performed with a scan rate of 1°C.min^-1^ at 222 nm using a 1-mm path-length cuvette. The Tm value was the temperature at the midpoint of the unfolding transition and was determined by sigmoidal fitting of the unfolding transition. The effect of adenosine nucleotides (200 μmol.L^-1^) and/or Mg^2+^ (200 μmol.L^-1^) on the mortalin structure was also investigated using thermal-induced unfolding followed by CD_222 nm_. Both stock solutions of these ligands were prepared in TKP buffer.

The intrinsic fluorescence emission measurements were performed in an F-4500 fluorescence spectrophotometer (Hitachi) using a 10×2-mm path-length cell with mortalin (1–2 μmol.L^-1^) in TKP buffer at room temperature. The excitation wavelength (λ) was set to 295 nm with a bandpass of 4 nm, and the fluorescence emission was measured from 310 up to 420 nm with a bandpass of 4 nm. The data were analyzed using the maximum fluorescence emission wavelength (λ_max_) and spectral center of mass (<λ>), as previously described [34, 37], with a wavelength between 320 and 380 nm. The effect of the temperature on the mortalin structure was also followed by fluorescence emission. The excitation λ was set to 295 nm, and the emission spectra were collected between 310 and 420 nm after 4 min of equilibration at each temperature. The data were analyzed by the <λ>-values as a function of the temperature.

### Hydrodynamic characterization

The Superdex 200 GL 10/30 column (GE Healthcare Life Sciences) equilibrated with TKP buffer (pH 7.5) was used to perform the aSEC experiments and to determine the mortalin Stokes radius (R_s_), as previously described [34]. The standard protein mixture was constituted by apoferritin (480 kDa/R_s_ 67 Å), γ-globulin (160 kDa/R_s_ 48 Å), BSA (67 kDa/R_s_ 36 Å), ovalbumin (45 kDa/R_s_ 29 Å), carbonic anhydrase (29 kDa/R_s_ 24 Å) and cytochrome C (12 kDa/R_s_ 14 Å) (Sigma-Aldrich). The frictional ratio (ƒ/ƒ_0_) was estimated by the ratio of the experimental R_s_ to the radius of a sphere of the same mass [39].

Analytical ultracentrifugation experiments were performed in a Beckman Optima XL-A analytical ultracentrifuge. The sedimentation velocity experiments for mortalin were conducted at concentrations from 300 up to 580 μg.mL^-1^ in TKP buffer at 7°C and 30,000 rpm (AN-60Ti rotor), and the data acquisition was performed at 236 nm. The SedFit software (Version 12.1, [40]) was used to fit the absorbance *versus* cell radius data, which yielded a continuous c(S) distribution function of the sedimentation coefficients. The frictional ratio (ƒ/ƒ_0_) parameter acted as a regularization parameter. The standard sedimentation coefficients (s_20,w_) were identified as the maximum of the peaks of the c(S) curves after corrections to eliminate the interferences caused by the buffer viscosity and density and by the temperature. The SedFit software was used to estimate the s_20,w_-values because the buffer viscosity (*η* = 1.0 × 10^-2^ poise), density (*ρ* = 0.99823 g.mL^-1^) and mortalin partial-specific volume (V_bar_ = 0.7336 mL.g-1) were supplied by the Sednterp program. Using the s_20,w_ at each protein concentration, we calculated the standard sedimentation coefficient at a protein concentration of 0 mg.mL^-1^ (s^0^_20,w_), which is an intrinsic parameter of the particle [41], through linear regression.

### Isothermal titration calorimetry

The interaction of mortalin with adenosine nucleotides (ATP and ADP) was assessed by ITC using an iTC200 microcalorimeter (GE Healthcare Life Sciences). Mortalin and adenosine nucleotides, at the indicated concentrations, were prepared in TKP buffer containing 2 mmol.L^-1^ Mg^2+^. Twenty-five 1.5-μL aliquots of adenosine nucleotides at concentrations from 200 to 250 μmol.L^-1^ were injected into 203.8 μL of 10–15 μmol.L^-1^mortalin at 20°C. The apparent enthalpy change for each injection was calculated by integrating the area under the peaks of the recorded time course of the power change. The heat associated with the injectant dilution was determined from the baseline at the end of the titration and subtracted from the data. The data were analyzed by the Microcal Origin software using the One Set of Sites curve-fitting model to calculate the apparent binding enthalpy change (ΔH_app_), binding stoichiometry (n), and association constant (K_A_). The apparent Gibbs energy (ΔG_app_) and apparent binding entropy change (ΔS_app_) were calculated using the following equation:

$$\Delta G_{app}=-RT\ln K_{A}=\Delta H_{app}-T\Delta S_{app}$$ (1)

### SAXS experiments

Small-angle X-ray scattering experiments were performed at the Brazilian Synchrotron Light Laboratory (LNLS, Campinas-SP, Brazil) using a monochromatic X-ray beam (λ = 1.488 Å) of the D02A-SAXS1 beamline. The sample-to-detector distance was ∼1000 mm, which corresponds to the scattering vector range of 0.015<q<0.35 Å^-1^, where q is the magnitude of the q-vector defined by q = (4π/λ)sinθ (2θ is the scattering angle). The mortalin samples were placed in a 1-mm path-length cell formed by two mica windows, and the scattering curves were recorded at 0.6 mg.mL^-1^ in TKP buffer. The samples and buffers were subjected to X-ray frames of 100 s, and the scattering curves were corrected for the detector response and scaled by the incident beam intensity and the sample’s attenuation. Tests with sequential frames were employed to check for radiation damage. The corrected scattering sample was subtracted from the scattering buffer curve. All of the intensities are in the absolute scale (cm^-1^), and this calibration was established from the scattered intensity of ultrapure water, which depends on the isothermal compressibility and on its electron density (*I*(0)_water, 293K_ = 0.01632 cm^−1^) [42]. The I(0) value is related to the protein concentration and MM and consequently with the monodispersity of the system.

The GNOM program was used to generate the p(r) curves using the experimental scattering curve. Using the p(r) function, it was possible to apply the DAMMIN program [43], which uses a simulated annealing optimization routine to search for a space-filling bead model (dummy atom model) that generates the best fit to the experimental scattering curve to obtain *ab initio* models for mortalin. We are aware that *ab initio* modeling does not provide a unique solution; thus, we performed the protein shape reconstruction by averaging 20 different *ab initio* models using the DAMAVER program package [44].

The final *ab initio* model constructed for mortalin from the SAXS data after its merge using the DAMAVER package was analyzed using the HydroPro software [45] to predict the hydrodynamic properties. This analysis allowed the comparison of the predicted hydrodynamic properties of the *ab initio* model to the obtained experimental data. The HydroPro software was set up with the radius of the atomic elements of 3.1 Å, with sigma factors of 8 and a minibead radius in the range of 4 to 2.0 Å. The parameters of MM (70.695 kDa) and V_bar_ (0.7357 cm^3^.g^-1^) were estimated from the amino acid sequence of mortalin using the Sednterp software. Parameters such as ρ and η (for standard conditions) were estimated using the Sednterp software at a temperature of 20°C.

Moreover, the experimental SAXS curve of mortalin could also be represented by a sum of two different protein conformations. In such data analysis the experimental scattering intensity is described as the weighted sum of the *E. coli* DnaK crystallographic structure bound to ATP (in the open conformation—PDB acc. n. 4B9Q [46]) and the NMR structure for *E. coli* DnaK in the ADP state (in the closed conformation—PDB acc. n. 2KHO [47]) form factors. The form factors as well as the respective weights were calculated and fitted to the experimental SAXS curve using GENFIT software [48, 49].

### ATPase activity

The mortalin and Hsp70–1A ATPase activity measurements were performed spectrophotometrically using the EnzChek Phosphate Assay kit (Invitrogen), as previously shown [50]. Summarily, the method allows the quantification of the inorganic phosphate (Pi) released from ATP hydrolysis by the enzyme. Mortalin (2.50 μM) and Hsp70–1A (2.25 μM) were prepared in TKP buffer (without phosphate ions and with 2 mmol.L^-1^ Mg^2+^) and incubated with ATP (0 to 2 mmol.L^-1^) for 90 min at 37°C. The negative control was in the absence of the enzyme. The samples containing the Pi hydrolyzed from ATP were incubated with 0.2 U of purine nucleoside phosphorylase (PNP) and 0.2 μmol.L^-1^ MESG, a chromogenic substrate, for 30 min at 23°C. The absorbance was measured at 360 nm. The amount of Pi released per minute (i.e., V_0_ in μmol.L^-1^.min^-1^) was plotted against the ATP concentration (mmol.L^-1^), and a Michaelis-Menten fitting was used (in the Origin software) to obtain the kinetic parameters. The k_cat_ was calculated by the ratio of V_max_ with the protein concentration used in the experiment. The specific activity (pmol min^-1^.μg^-1^) represents the amount of Pi released per time and mass of total protein. The stimulation effect of a client protein on the mortalin and Hsp70–1A ATPase activity was tested in the presence of the NR peptide (NRLLLTGY). It was pre-incubated with mortalin or Hsp70–1A in the presence of 2 mM of MgCl_2_ for 15 min at room temperature prior the incubation with 1 mM ATP for 90 min at 37°C, and then the Pi released was measured as aforementioned.

## Results

### Recombinant human mortalin was produced in soluble form and purified until homogeneity

**Fig 1:**
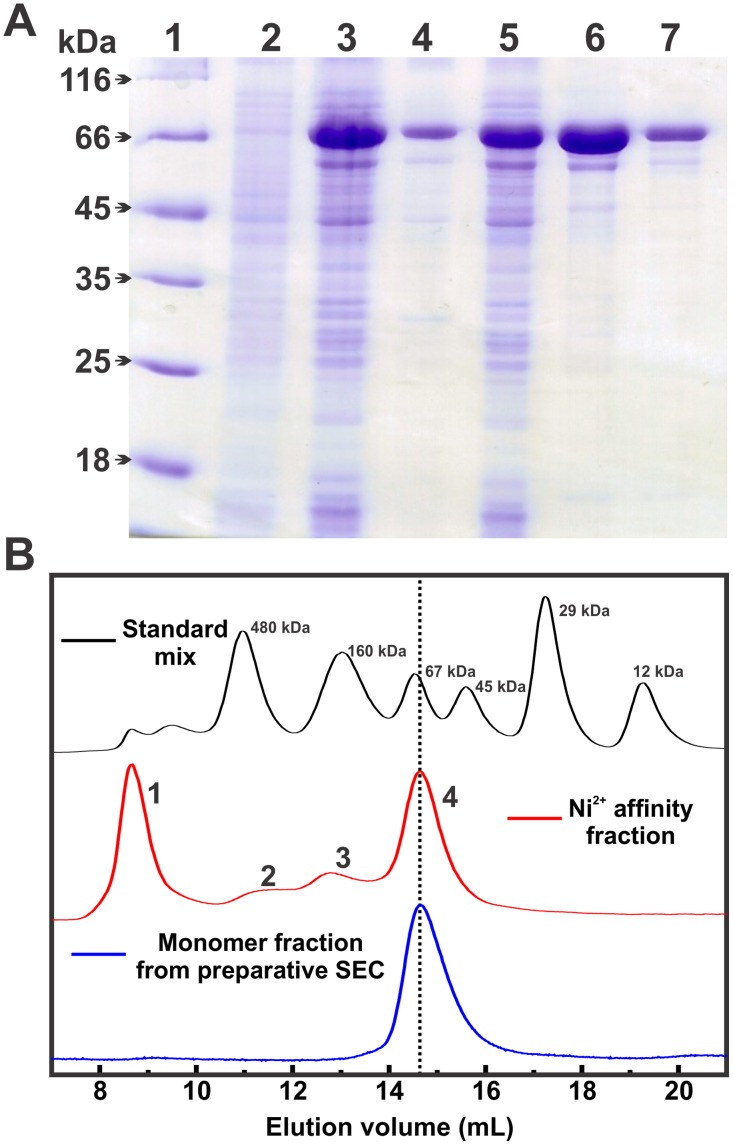
Mortalin production and isolation. Recombinant human mortalin (pET28a::mtHsp70) was co-expressed with recombinant hHep1 (pET23a::hHep1) in *E. coli* BL21(DE3) cells. **A**) SDS-PAGE of the produced and purified recombinant mortalin. 1) MM markers in kDa (left); 2) non-induced bacterial pellet; 3) induced bacterial pellet; 4) pellet of lysed cells; 5) supernatant of lysed cells; 6) fraction obtained from Ni^2+^ affinity chromatography; and 7) preparative SEC fraction. The final purity of mortalin was higher than 95%. **B**) aSEC profile of mortalin after Ni^2+^ affinity chromatography (red line), which showed that mortalin was eluted into four fractions (see text for details). The monomeric fraction (4) was immediately reloaded into the aSEC column and eluted as a monomer (blue line). The standard protein mixture profile is represented by the black line, and the MM of each protein is shown. The vertical dashed line marks the monomeric mortalin elution volume.

The recombinant human mortalin was produced in a soluble state through its co-expression with hHep1, as previously shown [34]. The purification of mortalin to the monomeric state was performed through two chromatography steps intercalated by its incubation with alkaline phosphatase to eliminate any traces of adenosine nucleotides [37]. Fig. 1A depicts the production and purification steps. The obtained mortalin was more than 95% pure and a monomeric species. For unclear reasons, the hHep1 produced by the pET23a expression vector had low yield (data not shown) [32] and was not easily detected by SDS-PAGE (Fig. 1A). However, its effect on the maintenance of human mortalin in the soluble fraction of the lysed cells was similar (data not shown) to that obtained when hHep1 was expressed by the pQE2 expression vector [34].

Fig. 1B shows a typical aSEC profile, which shows that mortalin was eluted as four main species: 1) an aggregate species eluted into the column void, 2) a possible tetrameric species, 3) a dimeric species, and 4) the main monomeric species, which is described as the Hsp70 active form [37, 51]. The fraction corresponding to the mortalin monomeric species was isolated and concentrated to 0.6–1.0 mg.mL^-1^ (8–14 μmol.L^-1^) for biophysical/biochemical characterization. When this fraction was immediately reloaded into the aSEC column, mortalin behaved as a monodisperse monomeric species, but it self-associated or aggregated under storage conditions (>24 h) and at high protein concentrations (> 1.0 mg.mL^-1^) (data not shown).

### Human mortalin was obtained in a folded state

**Fig 2:**
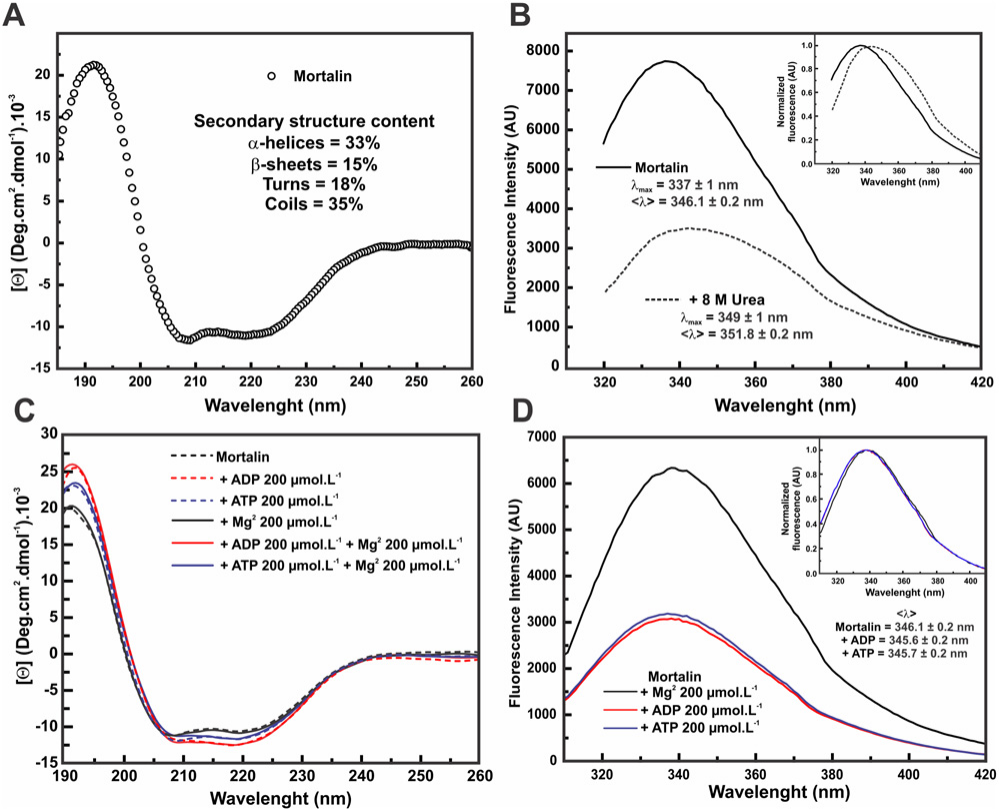
Mortalin was produced in its folded state. **A**) The CD spectrum shows that mortalin was obtained with a secondary structure composed of both α-helices and β-sheets. The secondary structure content, which was estimated by the CDNN deconvolution, is depicted into the figure (error < 5%). **B**) The intrinsic fluorescence emission (excitation λ at 295 nm) spectrum of mortalin had a λ_max_ value of 337 ± 1 nm and a <λ> value of 346.1 ± 0.2 nm, indicating that the single Trp residue was at least partially buried. In the presence of 8 mol.L^-1^ urea, the spectrum was quenched and suffered a red shift (λ_max_ of 349 ± 1 nm and <λ> of 351.8 ± 0.2 nm), suggesting unfolding. *Inset*: normalized spectra. **C**) Mortalin CD spectra in the presence of the indicated ligands. **D**) Intrinsic fluorescence emission (excitation λ at 295 nm) spectra of mortalin in the presence of the indicated ligands, which led to a suppression of the fluorescence emission intensity and to a slightly blue shift in the spectra, as shown in the normalized fluorescence spectra (*inset*). These results suggested that mortalin was expressed in both its folded and functional state. Moreover, the presence of adenosine nucleotides and Mg^2+^ ions led to slight conformational changes in the mortalin structure.

The secondary and tertiary structures of mortalin were investigated through CD and tryptophan fluorescence emission, respectively. Fig. 2A represents the mortalin CD spectrum corrected to [Θ] with two minima (at 208 and 222 nm) and a maxima (at 192 nm), which are characteristic of proteins containing a large portion of α-helices in their secondary structure. Using the CDNN Deconvolution program for deconvoluting the CD spectrum, we assessed the mortalin secondary structure content as follows: 33% of α-helices, 15% of β-sheets, 18% of turns and 35% of coils (errors < 5%). Similar values were previously reported for human Hsp70–1A [37] and other Hsp70 proteins [52, 53].

Mortalin has a single tryptophan residue located in the NBD near the nucleotide-binding pocket (data not shown). Therefore, intrinsic fluorescence experiments can provide reliable information on the burial of this residue and consequently the compactness of the tertiary structure in the surrounding region. The mortalin fluorescence emissions recorded under the native and chemically denaturated conditions are presented in Fig. 2B. Under the native conditions, the λ_max_ and <λ> values observed were of 337 ± 1 nm and 346.1 ± 0.2 nm, respectively. In the presence of 8 mol.L^-1^ urea, mortalin appears to unfold because the findings revealed fluorescence suppression and a spectrum red-shift (Fig. 2B, *inset*) in such a way that the λ_max_ and <λ> values changed to 349 ± 1 nm and 351.8 ± 0.2 nm, respectively. These data suggest that the single tryptophan present in the mortalin structure is located in an environment that is partially protected from the solvent.

The effect of adenosine nucleotides (ADP or ATP) and Mg^2+^ ions was also investigated by CD and fluorescence techniques. The presence of these ligands led to slight changes in the CD spectra (Fig. 2C) and to fluorescence emission suppression (Fig. 2D) with slight changes in the λ_max_ and <λ> values (Fig. 2D, *inset*). Altogether, these results indicate that mortalin was obtained in the folded state and that the presence of adenosine nucleotides led to slight conformational changes in both the secondary and local tertiary structures, suggesting that these ligands interacted with mortalin.

### The mortalin monomer was slightly elongated

**Fig 3:**
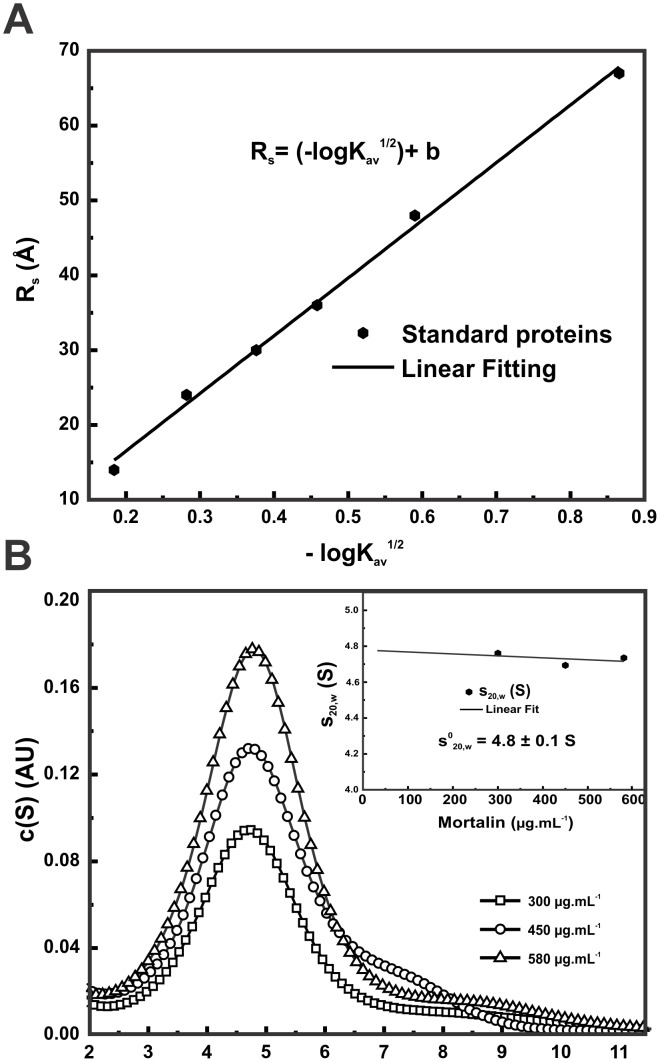
Monomeric mortalin was slightly elongated in solution. **A**) Estimation of the mortalin R_s_ based on the aSEC data presented in Fig. 1B. The graph depicts the R_s_ of standard globular proteins as a function of the partial coefficient k_av_, which yielded an R_s_ value for mortalin of 35 ± 2 Å. **B**) Sedimentation velocity data resulting from the c(S) distribution of mortalin, which behaved mainly as a monomeric species of 76 ± 4 kDa with ƒ/ƒ_0_ of approximately 1.36 ± 0.01 under the tested conditions (see Materials and Methods section). *Inset*: Determination of the s^0^_20,w_ of mortalin through the dependence of s_20,w_ on the protein concentration. Mortalin had a s^0^_20,w_ value of 4.8 ± 0.1 S (Table 1).

Through hydrodynamic techniques, some conformational features of the mortalin monomeric fraction were evaluated. Using the elution volume of the mortalin depicted in Fig. 1B, it was possible to estimate its R_s_ through the dependence of the R_s_ of standard proteins and the partial coefficient k_av_ (Fig. 3A). The R_s_-value determined for mortalin was 35 ± 2 Å. Using a theoretical hydrodynamic radius of 27 Å, which was calculated for a non-hydrated spherical particle with the same size as monomeric mortalin, we estimated a ƒ/ƒ_0_ value of approximately 1.3 (Table 1), which suggests that mortalin as a monomer possesses a slightly elongated shape.

**Table 1:** Summary of the hydrodynamic and structural data of mortalin.

**Mortalin structural and hydrodynamic properties**	**Predicted for a sphere^†^**		**Experimental determination**			
	**Monomer**	**Dimer**	**aSEC**	**AUC^#^**	**SAXS**	**HydroPro**
MM (kDa)	70.7	141.4	75 ± 4	76 ± 4	66 ± 2	-
*s^0^_20,w_* (S)	6.1	9.6	-	4.8 ± 0.1*	-	4.9 ± 0.2
R_s_ (Å)	27	34	35 ± 2	-	-	34 ± 3
ƒ/ƒ_0_	-	-	1.30^¥^	1.36 ± 0.01	-	1.30
R_g_ (Å)	-	-	-	-	36 ± 2^§^ 38 ± 2^&^	33 ± 2
D_max_ (Å)	-	-	-	-	130 ± 10	116 ± 3

^†^ Values predicted as globular monomers in water and 20°C (predicted by the Sednterp software)

^#^ calculated from SedFit from the sedimentation velocity data;

* data extrapolated for water, 20°C and 0 mg.mL^-1^ of protein;

^§^ data from Guinier law;

^&^ data from p(r) curve;

^¥^ data obtained using the Stokes equation for proteins of known Rs (predicted by Sednterp software).

To further investigate the hydrodynamic properties of mortalin, we performed sedimentation velocity AUC experiments. Fig. 3B presents the c(S) distribution function obtained for the mortalin monomeric fraction. This species behaved mainly as a monomer of 76 ± 4 kDa, with an s^0^_20,w_-value of 4.8 ± 0.1 S (Fig. 3B—*inset*) under the tested conditions. The ƒ/ƒ_0_–value supplied by the SedFit software was 1.36 ± 0.01 (Table 1), which is in agreement with that observed by the R_s_/R_0_ ratio and corroborates the slightly elongated shape of the mortalin monomeric species. Both the ƒ/ƒ_0-_ and s^0^_20,w_-values are in agreement with the values reported for human cytosolic Hsp70–1A [51], suggesting their similar shapes. However, a shoulder was also observed in the c(S) distribution function of mortalin, suggesting the presence of oligomeric species, which may have appeared due to the long sedimentation velocity run. The hydrodynamic data confirmed that mortalin was purified mainly in its monomeric fraction and indicated that it has a slightly elongated shape. The complementarity of the results found with different techniques is noteworthy, suggesting that artifacts are likely not present.

### Mortalin has slightly higher ATPase activity than Hsp70-1A

**Fig 4:**
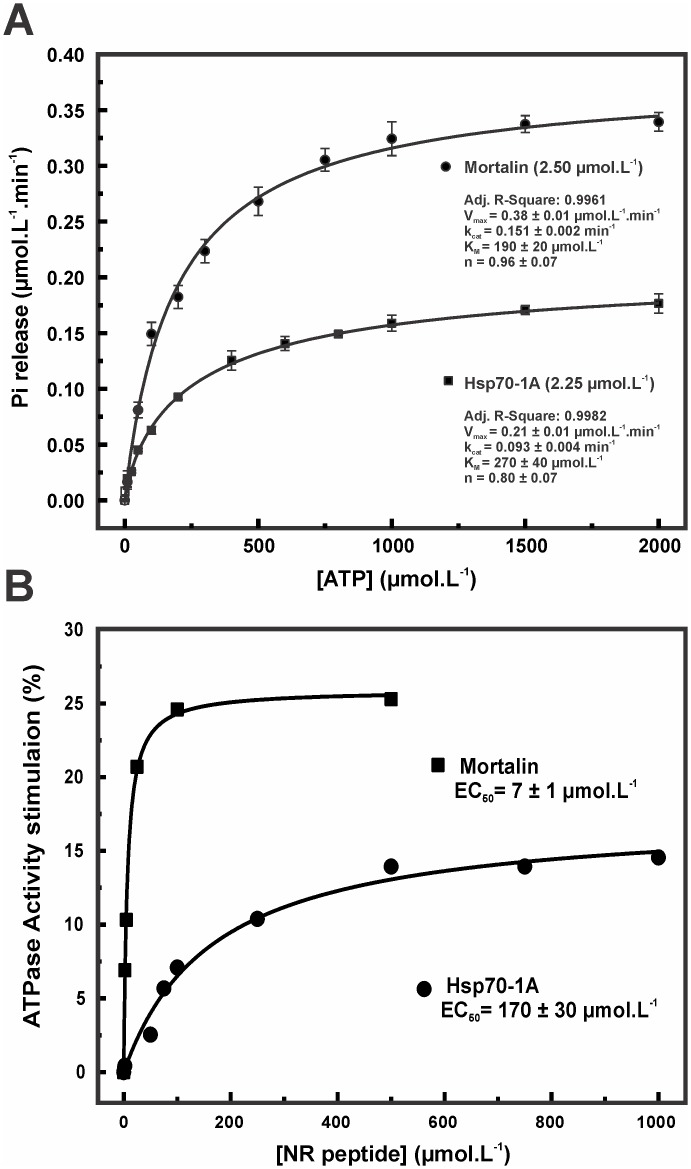
Mortalin has higher ATPase activity than Hsp70–1A. **A**) Mortalin (2.50 μmol.L^-1^) and Hsp70–1A (2.25 μmol.L^-1^) were incubated with ATP (0–2 mmol.L^-1^) for 90 min at 37°C, and the Pi released as a result of ATP hydrolysis was quantified. The data were treated through Michaelis-Menten fitting for determination of the kinetic parameters, which are presented in the Figure and Table 2. The results suggested that both mortalin and Hsp70–1A exhibit low ATPase activity. Despite these findings, based on the k_cat_ value, mortalin presented higher ATPase activity than Hsp70–1A, although the K_M_ values of both are similar. **B**) Relative ATPase activity stimulation. Effect of the NR peptide titration on the basal ATPase activity of mortalin and Hsp70–1A at 1 mmol.L^-1^ ATP.

As previously mentioned, Hsp70 presents ATPase activity mediated by NBD, which is characterized by its weakness [54]. Here, we present the ATPase activity of mortalin and Hsp70–1A in the absence of any co-chaperones. Fig. 4A depicts the ATPase activity as a function of the ATP concentration and the Michaelis-Menten fitting for both mortalin and Hsp70–1A. We can observe that mortalin exhibits slightly higher ATPase activity than Hsp70–1A, as indicated by the molecular turnover numbers (k_cat_) of 0.151 ± 0.002 min^-1^ and 0.093 ± 0.002 min^-1^, respectively (Table 2). Despite this finding, both proteins present a low rate of ATP hydrolysis. Table 2 presents different values for the ATPase activity available in the literature for several Hsp70s. One can observe that both mortalin and Hsp70–1A presented rates of ATP hydrolysis that were similar to those of other Hsp70s, and this fact is absolutely relevant because they indicate regulation by co-chaperones, client proteins and other ligands [22]. Specifically, the Hsp70 activity cycle is regulated by the ATP/ADP-dependent bidirectional heterotrophic allostery present on NBD and by the client protein bound to the PBD, which controls the cycle. To test if mortalin and Hsp70–1A were allosterically actives, we used the NR peptide as stimulation factor. Fig. 4B depicts the relative ATPase activity observed for mortalin and Hsp70–1A at increasing NR peptide concentrations. For mortalin, a maximum of 25% of stimulation was reached at 100 μmol.L^-1^ NR peptide indicating a half maximal effective concentration (EC_50_) of 7 μmol.L^-1^ NR peptide. The NR peptide stimulated the Hsp70–1A ATPase activity by around 15%, which was reached at 500 μmol.L^-1^ NR peptide, resulting in EC_50_ of 170 μmol.L^-1^ NR peptide. These results indicate that both proteins were obtained allosterically actives. However, the differences in the EC_50_ values and maximum stimulation indicate that mortalin and Hsp70–1A should present slightly differences in client protein specificities. In addition, this activity cycle can also be regulated by co-chaperones, such as J-domain proteins (or Hsp40), which act by speeding up ATP hydrolysis, and nucleotide exchange factors (NEFs), such as GrpE proteins [22].

**Table 2:** Kinetic constants determined for human mortalin and human Hsp70–1A compared with those of homologous Hsp70.

**Protein**	**Specific activity (pmol.min^-1^.μg^-1^)**	**K_M_ (μmol.L^-1^)**	**k_cat_ (min^-1^)**	**Temperature (°C)**	**Ref.**
***E. coli*** **DnaK**	-	-	0.087 ± 0.007	37	[54]
			0.15	37	[65]
***E. coli*** **Hsc66**	-	12.7	0.083	23	[66]
**Bovine Hsc70**	1.14	1.37	0.15	37	[67]
**Rat Hsc70**			0.12		[68]
***Chlamydomonas reinhardtii* Hsp70B**	~ 9	118	-	30	[69]
**Yeast Ssa**	-	0.11 ± 0.04	0.031 ± 0.004	37	[70]
**Yeast Ssb**	-	147 ± 42	0.81 ± 0.13	37	[70]
**Human Hsp70–1A**	0.62 ± 0.02	270 ± 40	0.093 ± 0.002	37	[71]—This work
**Human Mortalin**	0.86 ± 0.02	190 ± 20	0.151 ± 0.002	37	This work

Comparing the k_cat_ to other members of Hsp70 family we notice that the values are too close of each other, Sadis & Hightower [67] found a k_cat_ of 0.15 min^-1^ for bovine brain Hsc70, while the recombinant rat Hsc70 presented 0.12 min^-1^ for the ATPase activity.

### Interaction studies with adenosine nucleotides by ITC

**Fig 5:**
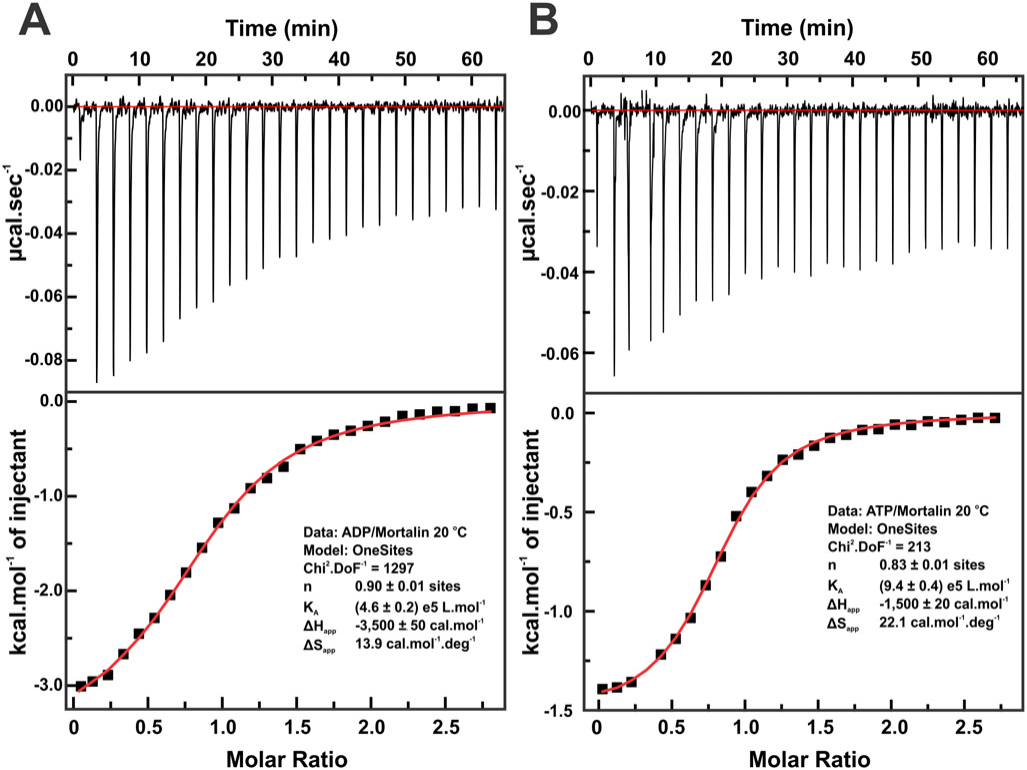
Mortalin interacts with ADP and ATP in a micromolar dissociation constant range. The mortalin interaction with ADP (**A**) and ATP (**B**) in the presence of Mg^2+^ was tested by ITC, suggesting K_Ds_ values of approximately 2.2 ± 0.1 μmol.L^-1^ and 1.1 ± 0.1 μmol.L^-1^, respectively. Moreover, the ITC data suggested that the interaction was directed by both enthalpy and entropy. *Upper panel*: The heat released at each ADP or ATP titration is presented by the negative peaks. The red line represents the baseline. *Lower panel*: The ΔH_app_ values were calculated by the integrated area of each ADP or ATP titration peak of the upper panel and plotted against the ADP/mortalin molar ratio. The red line represents the fit obtained by the one-site-binding model provided by the Origin program supplied with the ITC device in both cases. The fitting parameters are shown.

The ITC technique was used to investigate the interaction of mortalin with adenosine nucleotides. Fig. 5A (upper panel) presents the results of a calorimetric titration with ADP (~250 μmol.L^-1^), which reveal an exothermic profile. The apparent heat per mol of ADP injected (ΔH_app_) against the molar ratio of ADP/mortalin is depicted in the lower panel of Fig. 5A. The fitting of this curve indicated an exothermic ΔH_app_ of-3,500 ± 50 cal.mol^-1^ and a K_A_ value of 4.6 ± 0.2 × 10^5^ L.mol^-1^, suggesting a K_D_ of 2.2 ± 0.1 μmol.L^-1^. Based on the K_A_ and ΔH_app_ values and Equation 1, the ΔS_app_ value was calculated to be +13.9 cal.mol^-1^.deg^-1^. The interaction of ATP with mortalin by ITC is depicted in Fig. 5B. Similarly, the fitting of the calorimetric titration of ATP into mortalin solution yielded an exothermic ΔH_app_ of-1,500 ± 20 cal.mol^-1^ and a K_A_ value of 9.4 ± 0.4 × 10^5^ L.mol^-1^, suggesting a K_D_ of 1.1 ± 0.1 μmol.L^-1^. Using these values and Equation 1, the ΔS_app_ value was estimated to be +22.1 cal.mol^-1^.deg^-1^. These data suggested that both enthalpy and entropy drive the mortalin interaction with both adenosine nucleotides, as was also observed for human cytosolic Hsp70–1A [37]. However, the latter showed higher affinity for ADP than ATP under similar conditions [37], whereas mortalin appears to have higher affinity for ATP than ADP. The higher Tm_1_ that mortalin presented in the presence of ATP-Mg^2+^ in comparison to ADP-Mg^2+^ (see below—Table 3) also indicated that this protein has higher affinity for ATP than ADP. Therefore, the ITC data suggest that mortalin interacts with both ADP and ATP with different affinities and that these interactions induce conformational changes and result in different thermodynamic signatures (ΔH_app_ and ΔS_app_).

**Table 3:** Summary of the Tm transitions determined to mortalin by CD_222 nm_ in the presence of adenosine nucleotides and/or Mg^2+^.

**Mortalin plus ligands (200 μmol.L^-1^)**			**Tm_1_ (°C)**	**Tm_2_ (°C)**
**ATP**	**ADP**	**Mg^2+^**		
-	-	-	40.1 ± 0.6	73.1 ± 0.9
+	-	-	41.4 ± 0.8	72 ± 1
-	+	-	40.4 ± 0.8	71.1 ± 0.6
-	-	+	40.5 ± 0.7	73.9 ± 0.8
+	-	+	46.4 ± 0.6	71.6 ± 0.5
-	+	+	43.7 ± 0.7	70 ± 1

Values obtained by averaging 4 independent preparations.

The stoichiometry of the interaction was approximately 0.8–0.9 for both ligands (Fig. 5), indicating a 1:1 stoichiometry because the NBD has one adenosine nucleotide-binding site. The value near 0.8 can be explained by a fraction of mortalin that is not responsive to binding ADP or ATP, likely due to the self-association process. In fact, the parallel analysis of mortalin samples at the end of the ITC experiments by aSEC showed the presence of 10–20% other species that are likely dimers (data not shown) due to the mortalin concentrations (~15 μmol.L^-1^) and the temperature used (20°C). The presence of other oligomeric species in the AUC experiments (presented here) at lower protein concentrations and experimental temperature conditions corroborate this hypothesis. When the protein concentration was corrected to simulate the mortalin monomeric fraction in the ITC sample (i.e., 80–90%), the stoichiometry reached almost 1 without causing changes in the values of the other thermodynamic parameters (data not shown). These observations noted the difficulties associated with working with mortalin in the concentrations and experimental conditions reported here and also suggested that the other mortalin oligomeric species either did not interact with adenosine nucleotides or did interact with a lower affinity constant.

### Structural analysis of mortalin by SAXS

**Fig 6:**
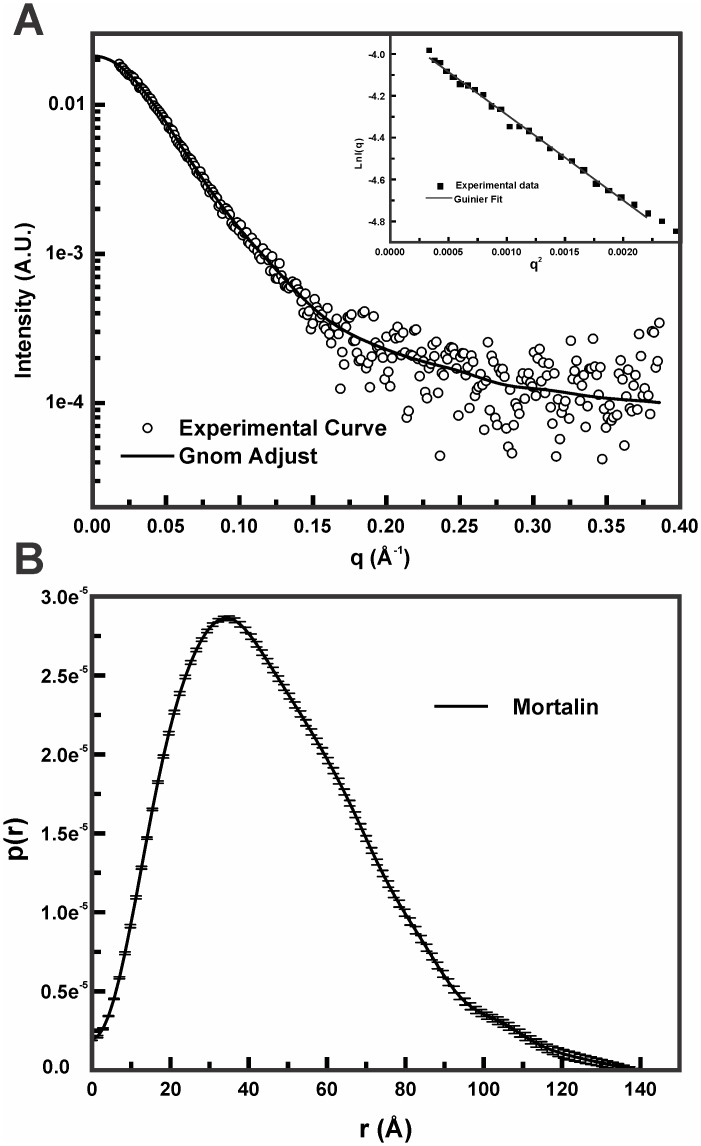
Mortalin has an elongated shape in solution. **A**) Experimental mortalin SAXS curve (open circles) suggesting that it behaved as a monodisperse system, as was confirmed by the evaluation of the Guinier region of the curve (*inset*). The GNOM fit is represented by a black line. Based on Guinier’ law (red line—*inset*), mortalin had a Rg value of 36 ± 2 Å (see text for details). **B**) The SAXS data were used to generate the p(r) distribution curve, which indicated that mortalin has a prolate shape and a D_max_ value of 130 ± 10 Å.

To obtain more information on the structure of mortalin in solution, we performed SAXS experiments. It is well known that some important parameters of the proteins can be directly obtained from the scattering curve, such as the radius of gyration, R_g_, and MM, using the I(q→0) of the scattering curve obtained from Guinier’s law (Fig. 6A—*inset*). Our data indicated that the R_g_ and MM values calculated for mortalin were 36 ± 2 Å and 66 ± 2 kDa, respectively. These results are in agreement with a monomeric and monodisperse mortalin (Table 1).

Fig. 6A shows the experimental scattering curve of mortalin along with the best curve obtained with the GNOM software, and the respective p(r) function can be appreciated in Fig. 6B. The p(r) curve noted that mortalin has a prolate shape with a maximum dimension (D_max_) of 130 ± 10 Å, whereas the protein R_g_ is 38 ± 2 Å, which is in accordance with the Guinier analysis.

**Fig 7:**
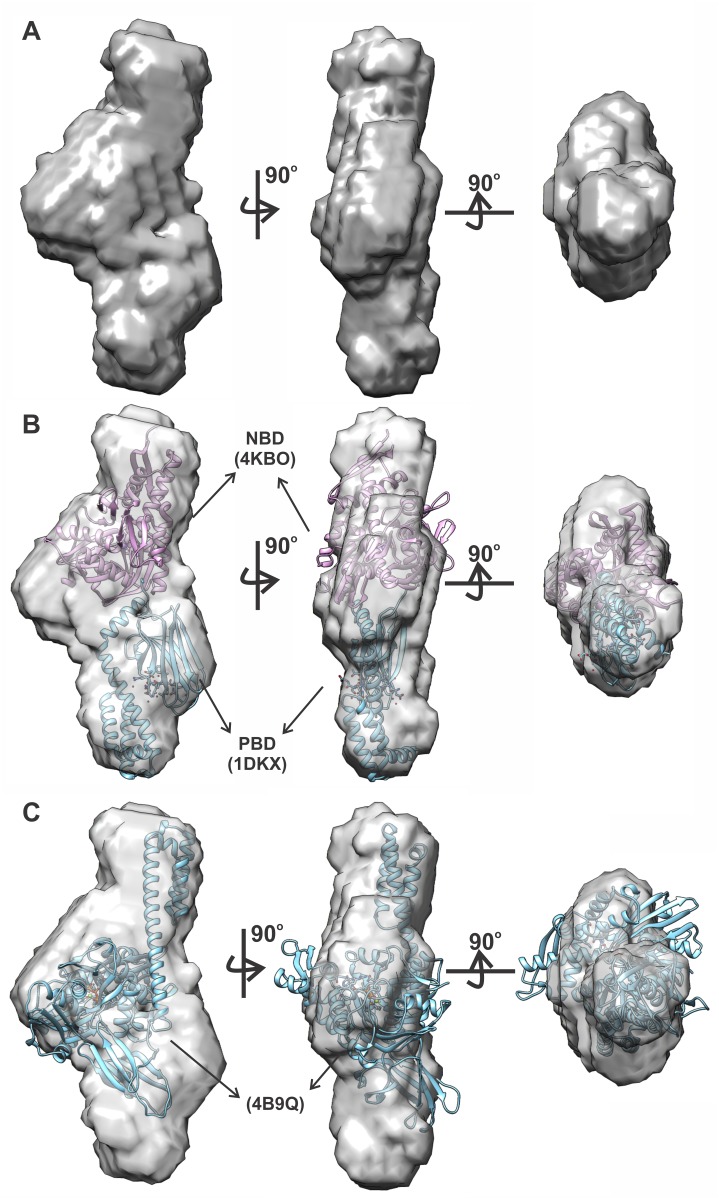
Mortalin *ab initio* models. The mortalin SAXS curve was used by calculating 20 low-resolution *ab initio* models using the DAMMIN software, and these were merged using the DAMAVER package. The result is the final *ab initio* model presented in several orientations (**A**). Manual superposition of the crystallographic structures of the mortalin NBD (PDB acc. no. 4KBO—magenta) and PBD of the *E. coli* DnaK (PDB acc. no. 1DKX—blue) into the *ab initio* model (**B**). Manual superposition of the crystallographic structure of the full length *E. coli* DnaK (PDB acc. n. 4B9Q—blue) into the mortalin *ab initio* model (**C**). Figures generated by the UCSF Chimera software (version 1.9).

Using the p(r) function, 20 independent *ab initio* models for mortalin in solution were generated. These 20 DAMMIM models were merged using the DAMAVER program, resulting in the final *ab initio* model (Fig. 7). The normalized spatial discrepancy (NSD) of the DAMMIM models was 0.7 ± 0.1, which indicated the overall quality of the generated models [55]. One should bear in mind, however, that this procedure is valid for monodisperse systems [56].

The final *ab initio* model was also subjected to HydroPro program analysis to estimate some of its hydrodynamic and structural properties (Table 1), which were close to the experimental ones. These results suggested that the proposed model based on the SAXS data analysis represents mortalin in solution. The *ab initio* model noted that mortalin has an elongated shape (Fig. 7A). Fig. 7B is the manual adjustment of the crystallographic structures of the mortalin NBD (PDB acc. no. 4KBO), obtained in apo-state [57], and PBD of the *E. coli* DnaK (PDB acc. no. 1DKX) bound to a synthetic peptide in the peptide binding site [58], into the *ab initio* model. This adjustment suggests that the *ab initio* model is suitable to accommodate both NBD and PBD of Hsp70 even considering that the model was generated from SAXS curve in apo-conditions. The full length *E. coli* DnaK crystallographic structure (PDB acc. n. 4B9Q), obtained bound to ATP and with the PDB in the open conformation [46], was used for its manual adjustment into mortalin *ab initio* model. As can be seen in Fig. 7C, in spite of the similar dimensions, the mortalin *ab initio* model did not properly adjust some portions of the crystallographic structure. This discrepancy is probably due to the protein dynamics in solution. It is noteworthy, in the SAXS point of view, the scattering curve is a weighted average of all protein conformations present in solution [56] and the *ab initio* model is a rigid representation of the scattering curve.

Moreover, it is not possible to neglect the possibility that mortalin solution structure can be represented by a combination of the *E. coli* DnaK in which the PBD is in the open (PDB acc. n. 4B9Q [46]) and in the closed (PDB acc. n. 2KHO [47]) structures. In order to check for such possibility, we used the full length DnaK structures and GENFIT software [48] to elucidate if the combinations of these structures are present in mortalin apo solutions. According to our data analysis, the mortalin SAXS curve was fitted supposing such possibility (data not shown), considering that almost 85% of the mortalin molecules were in the closed conformation, whereas 15% were in the open conformation. Therefore, in the conditions tested, mortalin behaves as equilibrium of, at least, two conformations where the PBD conformational equilibrium was dislocated to the closed conformation.

### Mortalin is formed by domains with different stabilities

**Fig 8:**
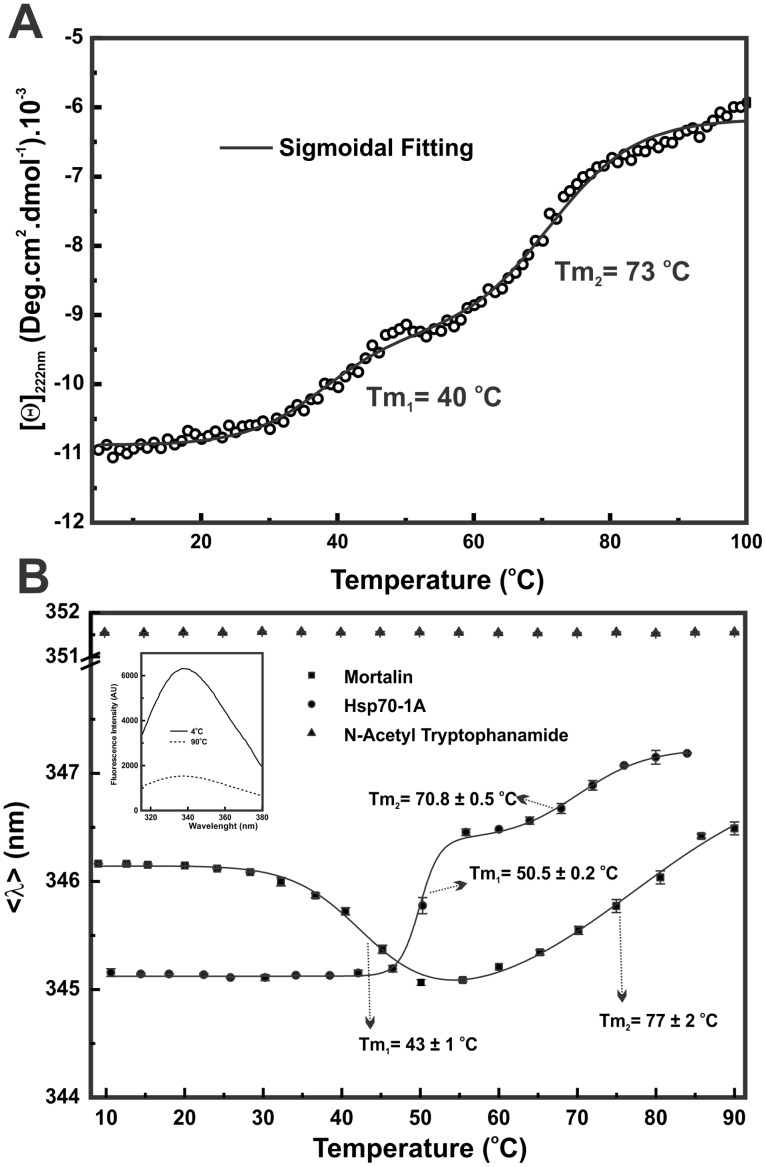
Mortalin is composed of at least two domains with different stabilities. **A**) The thermal-induced unfolding of mortalin followed by CD_222 nm_ presented two well-defined transitions with Tm values centered at 40 and 73°C; however, mortalin did not unfold completely (see text for details). **B**) Thermal-induced unfolding of mortalin followed by intrinsic fluorescence emission and represented as the <λ>-signal showing that mortalin suffered two blue-shift transitions with Tm values of approximately 43 and 77°C. Hsp70–1A presented two red-shift transitions with Tm values at 50.5 and 70.8°C. N-acetyl tryptophanamide at the same buffer conditions was used, as a control, and no transitions were observed at the <λ>-signal (351.5 ± 0.2 nm). The blue-shift transition suggested that mortalin associated or aggregated in the thermal-induced unfolding experiments (see text for details).

Through a similarity alignment to other Hsp70s, the mortalin primary structure points to two conserved domains [22]. To study the mortalin domain organization and stability, we made use of thermal-induced unfolding strategies. We followed both the secondary and tertiary structures of mortalin by CD and intrinsic fluorescence emission, respectively. Fig. 8A shows the data for mortalin thermal-induced unfolding followed by CD_222 nm_, which show at least two well-defined transitions. The first transition, which was represented by the loss of 20% of the CD_222 nm_ signal, had a Tm_1_ centered at 40°C. This value was 5–6°C less than that reported for the Tm_1_ of human cytosolic Hsp70–1A, which has three thermal transitions [37]. The second transition represented a forfeiture of approximately 30% and presented a Tm_2_ of approximately 73°C. Interestingly, the CD signal was maintained at approximately-5.000 deg.cm^2^.dmol^-1^ at 90°C, suggesting that mortalin could retain part of its secondary structure, even at high temperatures. Human cytosolic Hsp70–1A also has a discrete thermal transition centered at 68°C and a third transition at temperatures higher than 80°C [37]. Mortalin likely presents similar thermal induced unfolding in a number of events, but we did not observe the third transition by CD_222 nm_. Interestingly, *E. coli* DnaK thermal induced unfolding followed by CD_222nm_ also unfolding through two transitions with similar Tms to those observed for mortalin [59–61].

We also followed the thermal-induced unfolding of mortalin by fluorescence emission using the <λ>-signal as a probe (Fig. 8B). As expected, the temperature increment led to mortalin fluorescence emission quenching due to the water effects (Fig. 8B—*inset*). However, the mortalin <λ>-signal showed a blue-shift transition followed by a red-shift as a function of the temperature, whereas the <λ>-signal of the N-acetyl-tryptophanamide showed no change as a function of the temperature.

The thermal transitions observed for mortalin by fluorescence emission were centered at 43 and 77°C, respectively. Despite the Tms being slightly higher than those observed by CD_222nm_, the Tms were inside the experimental error, in agreement with the values observed by CD_222nm_, suggesting the correlation of these events in both the secondary and tertiary structures of mortalin. However, the Trp is located in just one of the domains (NBD), but it was apparently affected by the thermal unfolding of both domains or the unfolding of NBD in two transitions. We also performed the same experiment with Hsp70–1A and observed two red-shift transitions (Fig. 8B) with Tms that were similar to those reported for the thermal-induced experiments followed by CD_222 nm_ [37].

The blue-shift transition observed for mortalin promptly suggests that thermal-induced unfolding led to mortalin NBD packing and/or association/aggregation. This event may be mediated by the NBD because the single mortalin Trp is located in this domain. Therefore, the apo NBD of mortalin, under the conditions tested here, appears to be prone to aggregate at temperatures close to the physiological temperatures or in fever states. Interestingly, it has been reported that Hsp70 aggregates when it is thermally unfolded [52]. Specifically, it has been shown that mortalin has an aggregation tendency that is dependent on its NBD [32]. Taken altogether, these results indicate that mortalin has different domain stabilities and could unfold by a slightly different mechanism in comparison to Hsp70–1A.

The effect of adenosine nucleotides (200 μmol.L^-1^) and/or Mg^2+^ (200 μmol.L^-1^) on mortalin stability was investigated using thermal-induced unfolding followed by CD_222 nm_. The thermal-unfolding profiles in the presence of these ligands were similar (data not shown) to those found in the absence of these ligands (Fig. 8A), and Table 3 summarizes the Tms estimated in the absence and presence of different combinations of ligands. Surprisingly, the presence of Mg^2+^, ADP or ATP alone did not induce significant changes in either Tm_1_ or Tm_2_ (Table 3). These results contrast those observed for human cytosolic Hsp70–1A [37], where the adenosine nucleotides alone increased the first thermal-induced transition by approximately 4°C followed by CD_222 nm_ under similar conditions. Nonetheless, the combination of ATP-Mg^2+^ or ADP-Mg^2+^ increased the Tm_1_ of mortalin to 46°C and 44°C, respectively, resulting in an increment of 4–6°C in the Tm_1_ (Table 3). For human Hsp70–1A, the combination of adenosine nucleotides and Mg^2+^ ions also led to an additional increment in the first Tm of 7–9°C [37], indicating the importance of the Mg^2+^ ions for adenosine nucleotide binding. These results suggested that adenosine nucleotides bind to mortalin and stabilize the NBD structure.

## Discussion

In this manuscript, we report the production and purification of recombinant human mortalin in its monomeric and functional form without the use of unfolding/refolding strategies. Recombinant mortalin production was reached by its co-expression with the hHep1 co-chaperone, which maintains mortalin in the supernatant of the lysed *E. coli* cells [34]. For purification purposes, the mortalin was cloned into a pET28a expression vector, and hHep1 was cloned into a pET23a expression vector, which produces hHep1 in undetectable amounts (data not shown), as was reported by a previous study [32]. This allowed the production of mortalin samples with substoichiometric and/or undetectable amounts of hHep1, as attested by the SDS-PAGE figures and the MM determinations by aSEC, AUC and SAXS techniques.

To attest that mortalin was produced and purified in its functional state, enzyme kinetics experiments were performed to evaluate the basal ATPase activity of mortalin compared with that of Hsp70–1A. We observed that mortalin had a slightly higher ATPase activity than Hsp70–1A, but both proteins presented them in the same order of magnitude than other Hsp70s. We also observed that both proteins had K_M_ values in the higher μmol.L^-1^ range. We also tested if the ATPase activity of mortalin and Hsp70–1A was stimulated by the NR peptide. The results pointed out that mortalin and Hsp70–1A presented, at NR peptide saturation, increments of 25% and 15% on their basal ATPase activity, respectively, indicating that both recombinant proteins were produced in their allosterically forms. Nevertheless, they have showed some differences in client protein specificities since the EC_50_ registered for mortalin was approximately 25 times lower than for Hsp70–1A. The NR peptide has been used as client protein model for *E. coli* DnaK [58] and similar ATPase stimulation was also reached for this protein at 100 μmol.L^-1^ NR peptide [62]. These results also indicate that human recombinant mortalin is functionally similar to *E. coli* DnaK (see below).

ITC experiments were performed to test the interaction of mortalin with adenosine nucleotides. Because mortalin has weak ATPase activity, we used ATP in these experiments instead of a non-hydrolysable ATP analogue. The results indicated that mortalin interacts with ATP and ADP at a low micromolar K_D_ range and that the interaction is driven by enthalpy and entropy, as was also observed for human cytosolic Hsp70–1A [37]. In contrast to the latter, the interaction of mortalin with adenosine nucleotides was dependent on the presence of Mg^2+^ ions, as indicated by the thermal-induced unfolding experiments and the Tms (Table 3). Moreover, only two well-defined thermal transitions were observed for mortalin, whereas three transitions were reported for Hsp70–1A [37]. Therefore, these two proteins present different stabilities.

We characterized the recombinant mortalin by several biophysical tools and observed properties that are similar to those reported for other Hsp70 proteins [37]. It is constituted by α-helix and β-sheet secondary structures, as estimated by circular dichroism experiments. The single Trp residue located in the NBD is at least partially protected from the solvent, suggesting that this residue is buried in the protein hydrophobic interior. Our production strategy also allowed us to obtain mortalin in its monomeric form at least 24 h after purification by preparative SEC and at low protein concentrations. We observed that both storage at 4°C and high concentration induced mortalin to undergo self-association and/or aggregation processes (data not shown), which limits its study. Furthermore, the mortalin monomeric form has a slightly elongated shape, as attested by the aSEC, AUC and SAXS results. Human cytosolic Hsp70–1A was also studied by AUC and had a s^0^_20,w_ of approximately 4.4 S in the nucleotide-free state [51]. This value was slightly lower than the value observed for mortalin despite the similar MM and experimental conditions, which indicated that mortalin is slightly more globular than Hsp70–1A.

We also obtained SAXS data for mortalin in the monomeric state, which allowed the generation of a low-resolution model. The structural and hydrodynamic properties of this model were in accordance with the hydrodynamic and structural properties determined experimentally, indicating that the low-resolution model for mortalin was reliable. It is worth noting that the *ab initio* model suggests that the mortalin domains should be coupled to each other even in the absence of nucleotides because the central part of the model is enlarged in relation to the ends. Besides, the low resolution model obtained for mortalin from SAXS curve in apo conditions adequately accommodated the crystallographic structures of mortalin NBD and *E. coli* PBD, which resembles the ADP bound state (Fig. 7B). However, the mortalin *ab initio* model did not properly adjust the X-ray structure of *E. coli* DnaK bound to ATP, in which the PBD is in the open conformation. Actually, the mortalin SAXS curve was fitted regarding that it represents a conformational equilibrium between, at least two conformations, in which the PBD is in the open and closed conformations.

We employed unfolding strategies to monitor the domain organization of mortalin. Thermal-induced unfolding followed by CD_222 nm_ indicated that mortalin is composed of at least two domains because two well-determined transitions were observed. Interestingly, the first transition started at 35°C and had a Tm_1_ centered at 40°C in the nucleotide-free state. Thermal-induced unfolding followed by fluorescence noted a blue-shift transition at approximately the same temperature observed for CD_222 nm_. Altogether, these results suggested that mortalin can associate during its thermal-induced unfolding. Because the NBD is related to this thermal-induced unfolding transition, its participation in mortalin self-aggregation can be hypothesized. Previous studies indicated that the first transition of Hsp70 thermal-induced unfolding involves the partial unfolding of the NBD because the presence of adenosine nucleotides increased the first Tm [37, 52, 53], as attested in this study for mortalin. Zhai et al. [32] also reported that mortalin self-aggregation depends on the NBD. Interestingly, *E. coli* DnaK also shows two transitions in the thermal-induced unfolding experiments [59–61] with comparable Tms to those observed for mortalin, indicating that both proteins have domains with similar thermal stabilities. Actually, mortalin shares more identity with *E. coli* DnaK (59%) than with Hsp70–1A (48%), which can explain their similarities in thermal stabilities as well as the ATPase activity stimulation observed upon NR peptide titration.

Altogether, despite the challenges associated with the production of mortalin in its monomeric form and functional state, this work shed light on the mortalin structure and dynamics and indicates a strategy for the production of functional mortalin in sufficient amounts to perform additional functional studies. An allosteric pocket of Hsp70 proteins was recently identified as a promising site for structure-based drug design [63], and our study may help one to identify whether this binding site is also present in human mortalin and to test the interaction of mortalin with its specific inhibitor [29, 64]. Interaction studies of mortalin with inhibitor compounds and certain of its mitochondrial co-chaperones are underway.
